# Untargeted Metabolomics Uncovers Food Safety Risks: Polystyrene Nanoplastics Induce Metabolic Disorders in Chicken Liver

**DOI:** 10.3390/foods14162781

**Published:** 2025-08-10

**Authors:** Xuan Hu, Yinyin Liu, Yinpeng Ma, Jing Zhang, Lina Ma, Wanqiang Chen, Xiujun Tang, Junxian Lu, Lingzhi Chen, Guodong Cai, Jianchun Bian, Yushi Gao

**Affiliations:** 1 Jiangsu Institute of Poultry Science, Yangzhou 225009, China; 2 College of Veterinary Medicine, Yangzhou University, Yangzhou 225009, China; 3 Jiangsu Co-Innovation Center for Prevention and Control of Important Animal Infectious Diseases and Zoonoses, Yangzhou 225009, China

**Keywords:** nanoplastics, chicken, LC-MS/MS metabolomics, metabolic disruption, liver, food safety

## Abstract

Polystyrene nanoplastics (NPs) threaten agricultural ecosystems and the food chain; however, their hepatotoxicity in chickens, a key poultry species, remains unclear. This study investigated the effects of chronic NP exposure on hepatic metabolism to evaluate food safety risks in poultry products. Chickens were orally exposed to 100 nm polystyrene NPs via feed for 120 days. Histopathological evaluation, serum biochemical analysis revealed hepatotoxicity in NP-exposed poultry, characterized by histopathological liver injury, elevated lipid droplet accumulation, significantly increased alanine aminotransferase (ALT) activity, and elevated triglyceride (TG) levels (*p* < 0.05). Untargeted LC-MS/MS Metabolomics profiling identified 193 differentially abundant metabolites—predominantly organic acids and lipids—with L-leucine and NADH emerging as pivotal metabolic hubs. A KEGG pathway analysis demonstrated significant enrichment in purine metabolism and oxidative phosphorylation, while a gene set enrichment analysis (GSEA) confirmed the suppression of ABC transporters. Notably, the key biomarkers 9-cis-retinal and phenylalanyl phenylalanine were significantly altered, reflecting metabolic disturbances linked to NPs exposure. Overall, this study characterized exposure-associated metabolic signatures and established NP-induced hepatic injury phenotypes in poultry production systems.

## 1. Introduction

Microplastics (MPs, <5 mm) and nanoplastics (NPs, <1 μm) represent pervasive environmental contaminants that have infiltrated global food chains, posing unprecedented challenges to food safety [1]. The extensive use of plastic products, driven by their durability and versatility, has led to the accumulation of plastic waste that progressively fragments into MPs and NPs. These particles, particularly NPs, exhibit enhanced bioavailability due to their nanoscale size, high surface-area-to-volume ratio, and capacity to adsorb co-contaminants [2]. While substantial evidence exists for NPs contamination in aquatic ecosystems and seafood [3], terrestrial food systems, especially poultry production, remain critically understudied despite their centrality to human nutrition [4].

Data deficiency in terrestrial food chains: Current NPs research disproportionately focuses on marine environments and aquatic species [5]. Quantitative data on NPs translocation in poultry—a primary source of animal protein globally—are scarce, despite documented contamination of poultry feed and processing equipment with polyethylene (PE) and polyethylene terephthalate (PET) particles [6]. PE is introduced through multiple exposure pathways, including feed (e.g., equipment abrasion, packaging liners [5]), water distribution systems (plastic piping) [6]. Recent evidence confirms PE-derived residues in broiler muscle tissues at concentrations exceeding 700 mg/kg [5].

Mechanistic understanding of poultry toxicity: NPs have been detected in human blood [7], placenta [8], and breast milk [9], correlating with hepatic inflammation and metabolic dysfunction in mammalian models [7]. However, the hepatotoxic mechanisms of NPs in food-producing birds, particularly their impact on nutrient metabolism and residue accumulation in edible tissues, are virtually unexplored.

Risk assessment limitations: The absence of standardized biomarkers for NPs exposure in poultry hinders food safety monitoring. Regulatory frameworks currently lack NPs thresholds for animal feed and poultry products, creating significant policy gaps [8].

Growing evidence suggests that MPs may accumulate in various tissues and organs of terrestrial mammals, including the liver [9], kidneys [10], ovaries [11], testes [12], and others. The liver is a dynamic metabolic organ responsible for processing, metabolizing, and eliminating drugs and environmental toxins. Additionally, it serves as a primary location for the buildup of MPs within the body [13,14]. MPs with sizes between 4 and 30 μm have been found in human liver tissue. Previous studies have demonstrated that exposure to NPs can result in liver damage [15,16,17,18]. Exposure to MPs induces liver damage, inflammation, and even chronic liver disease (CLD) [19]. Therefore, it is of great significance to study the damage to the liver of living organisms. For poultry, an economically vital species occupying high trophic levels, hepatic NPs accumulation directly threatens food safety through potential toxin transfer and nutritional depletion [5]. The above studies suggest that acute MPs exposure exhibits a significant effect on the liver, but the effect of chronic MPs exposure on the liver is still unknown.

As an agriculturally important species occupying high trophic levels in terrestrial food chains, chickens represent a critical model for assessing NPs bioaccumulation risks and food safety implications. We hypothesized that chronic oral exposure to environmentally relevant doses of NPs disrupts hepatic metabolism in chickens, manifested through metabolite alterations, pathway dysregulation, and hepatotoxicity, ultimately compromising poultry product safety. Consequently, this study aims to identify exposure-associated biomarkers and provide foundational data for assessing NPs-related food safety risks in poultry products.

## 2. Materials and Methods

### 2.1. Ethics Statement

All experimental protocols in this study were reviewed and approved by the Ethics Committee for Animal Experiments of the Jiangsu Institute of Poultry Science (permit number: 202411001, Jiangsu Province, China). The animals were euthanized according to the American Veterinary Medical Association (AVMA)Guidelines for the Euthanasia of Animals (2020), ensuring full compliance with institutional and national guidelines for the care and use of laboratory animals.

### 2.2. NPs Characterization

Monodisperse NPs with a nominal diameter (diameter 100 nm, 10 mg/mL) were procured from Tianjin Baseline ChromTech Research Centre (Tianjin, China). The stock solution was aseptically diluted in pyrogen-free double-distilled water (ddH_2_O) to obtain working suspensions. Before every experiment, the NPs were sonicated for 10 min in a water bath at 40 kHz and 85% power. The NPs were oven-dried at 65 °C to powder, and the NPs morphology was observed using SEM (Gemini SEM 300, Oberkochen, Germany). The chemical characterization of NPs was performed using attenuated total reflectance FTIR spectroscopy (ATR-FTIR; Nicolet iS50, Thermo Fisher Scientific, Waltham, MA, USA). Post-acquisition processing, including atmospheric compensation and baseline correction, was performed using OMNIC v9.0 software (Thermo Fisher Scientific). Characteristic absorption bands of polystyrene were identified based on ASTM reference standards.

### 2.3. Experimental Animals

A total of 120 green-shelled egg chicken strain (120 days old; provided by JIPS) were acclimatized for 7 days under controlled environmental conditions (24 ± 1 °C, 55 ± 5% relative humidity) before random allocation into four experimental groups (*n* = 30 per group). NPs (100 nm diameter) were incorporated into standard feed at concentrations of 1, 10, and 100 mg/kg (designated as L-NPs, M-NPs, and H-NPs groups, respectively), while the control group received nanoparticle-free feed. The feeding trial lasted 120 days with ad libitum access to feed and water. Following a 12 h fasting period, chickens were humanely euthanized via CO_2_ asphyxiation. Liver tissues and whole blood were collected and immediately stored at −80 °C for subsequent metabolomic analysis and biochemical assessments, respectively.

### 2.4. Measurement of the Serum Biochemical

Serum was obtained by centrifugation of whole blood at 3000× *g* for 15 min at 4 °C. The serum alanine aminotransferase (ALT), aspartate aminotransferase (AST), and triglyceride (TG) levels were measured by fully automated instruments for measuring biochemical indicators (Beckman Coulter, AU480, Brea, CA, USA).

### 2.5. Hematoxylin–Eosin (HE) Staining

A uniform section of the liver was excised from each sample at the same anatomical location and fixed in neutral buffered formalin for 24 h. The tissue was subsequently dehydrated through a graded ethanol series, cleared in xylene, and embedded in paraffin wax. Sections were obtained using a microtome and mounted onto slides. Hematoxylin and eosin were used to stain the cell nucleus and cytoplasm of paraffin-embedded tissue sections that had been directly sectioned at a thickness of 5 µm. Every specimen was examined and documented utilizing a Leica light microscope DMI3000B (Leica, Wetzlar, Germany) outfitted with a digital camera.

### 2.6. Oil Red O (ORO) Staining

Fresh liver specimens were snap-frozen in Optimal Cutting Temperature (OCT) compound. A total of 5 μm cryosections to Oil Red O staining standardized protocol: Sections were incubated in 60% isopropanol (1 min), stained with freshly prepared Oil Red O working solution, differentiated in 60% isopropanol (30 s), rinsed briefly in distilled water, and counterstained with Mayer’s hematoxylin. The slices were observed in accordance with the aforementioned methodology (DMI3000B, Leica, Wetzlar, Germany).

### 2.7. Metabolomics Methodology

#### 2.7.1. Metabolite Extraction

Approximately 100 mg of tissue sample was homogenized in liquid nitrogen and subsequently resuspended in pre-chilled 80% methanol with thorough vortex mixing. After incubation on ice for 5 min, the homogenate was centrifuged at 15,000× *g* and 4 °C for 20 min. A portion of the supernatant was diluted with LC-MS-grade water to a final concentration of 53% methanol. The samples were then transferred to fresh microcentrifuge tubes and centrifuged again under the same conditions (15,000× *g*, 4 °C, 20 min). The resulting supernatant was collected and subjected to LC-MS/MS analysis.

#### 2.7.2. HPLC-MS/MS Analysis

Chromatographic separation was performed using a Vanquish UHPLC system (Thermo Fisher Scientific, Waltham, MA, USA) coupled with either an Orbitrap Q Exactive™ HF or HF-X mass spectrometer (Thermo Fisher Scientific). Samples were loaded onto a Hypersil Gold column (100 × 2.1 mm, 1.9 μm) at a flow rate of 0.2 mL/min with a 12 min linear gradient. The mobile phases consisted of (A) 0.1% formic acid in water and (B) methanol for positive ion mode, and (A) 5 mM ammonium acetate (pH 9.0) and (B) methanol for negative ion mode. The gradient program was as follows: 2% B (0–1.5 min), 2–85% B (1.5–4.5 min), 85–100% B (4.5–14.5 min), 2–100% B (14.5–14.6 min), and 2% B (14.6–16 min). Mass spectrometry parameters included a spray voltage of 3.5 kV, capillary temperature of 320 °C, sheath gas flow rate of 35 psi, auxiliary gas flow rate of 10 L/min, S-lens RF level of 60, and auxiliary gas heater temperature of 350 °C.

#### 2.7.3. Data Processing and Metabolite Identification

Raw data files from UHPLC-MS/MS were processed using XCMS for peak alignment, feature extraction, and quantification. Metabolite identification was achieved by matching experimental MS/MS spectra against high-quality spectral libraries with a mass tolerance of 10 ppm, considering relevant adducts. Background interference was removed by subtracting blank sample signals. Relative peak areas were calculated using the following normalization formula: Relative peak area = (Raw sample quantitation value)/(Sum of sample quantitation values/Sum of QC1 quantitation values). Metabolites exhibiting a coefficient of variation (CV) > 30% in QC samples were excluded. Data processing was conducted on a Linux operating system (CentOS 6.6) using R-3.4.3 and Python-3.5.0.

#### 2.7.4. Statistical and Bioinformatics Analysis

Metabolites were annotated using the KEGG, HMDB, and LIPID Maps databases. Multivariate statistical analyses, including principal component analysis (PCA) and partial least squares–discriminant analysis (PLS-DA), were performed using the meta-X software package. Univariate analysis (Student’s *t*-test) was applied to assess statistical significance (*p* < 0.05), with differential metabolites defined as those exhibiting variable importance in projection (VIP) scores > 1 and Fold Change (FC) ≥ 1.2 or ≤ 0.5.

Volcano plots were generated using ggplot2 in R, visualizing log2(FC) against −log10 (*p*-value). Cluster heatmaps were constructed with the Heatmaps package after z-score normalization. Pairwise correlations between differential metabolites were computed using Pearson’s correlation coefficient (cor() function in R), with significance assessed via cor.mtest() (*p* < 0.05 considered significant). Correlation matrices were visualized using the corrplot package. Pathway enrichment analysis was performed using the KEGG database, with metabolic pathways considered enriched when the ratio of annotated metabolites (x/n) exceeded the background ratio (y/N). Pathways with *p*-values < 0.05 were deemed statistically significantly enriched.

### 2.8. Statistical Analysis

All quantitative data are presented as the mean ± standard deviation (SD) from at least three independent biological replicates. Statistical analyses were performed using GraphPad Prism 9 (GraphPad Software, Inc., La Jolla, CA, USA). Intergroup differences were assessed using one-way analysis of variance (ANOVA), followed by Scheffé’s post hoc test for multiple comparisons. A *p* value < 0.05 was considered statistically significant.

## 3. Results

### 3.1. Characterization of Fluorescent NPs

**Figure 1:**
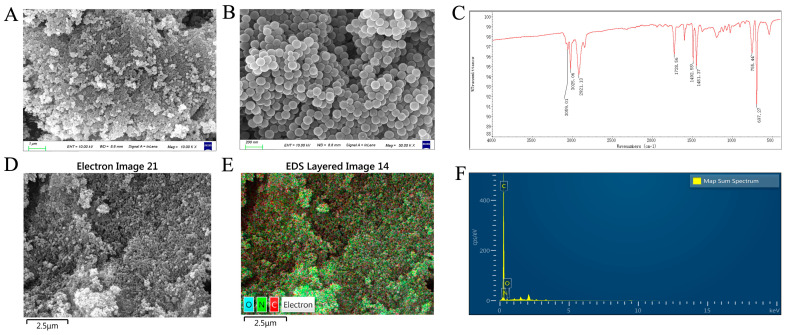
Morphological and chemical characterization of NPs. (**A**) SEM image of NPs (scale bar 1 μm). (**B**) High-resolution SEM image of NPs (scale bar 0.1 μm). (**C**) FT-IR spectrum of NPs displaying characteristic absorption bands. (**D**) Low-magnification SEM image of NPs (scale bar 2.5 μm). (**E**) Secondary electron SEM image of NPs (scale bar 2.5 μm). (**F**) EDS elemental analysis of NPs obtained using a SEM field-emission scanning electron microscope.

Scanning electron microscopy (SEM) characterized the NPs’ morphology. SEM analysis (Figure 1A,B,D–F and Table 1) revealed that the NPs were monodisperse, with smooth surfaces and a uniform spherical shape. The NPs solution exhibited homogeneous dispersion with well-defined boundaries, and particle sizes ranged from approximately 80 to 100 nm. Fourier transform infrared (FTIR) spectroscopy confirmed the molecular structure of polystyrene, with an absorption peak corresponding to the characteristic vibrational mode of the polystyrene benzene ring (Figure 1C).

### 3.2. The Influence of Exposed NPs on the Organ Coefficients and Morphology of Chicken Liver

**Figure 2:**
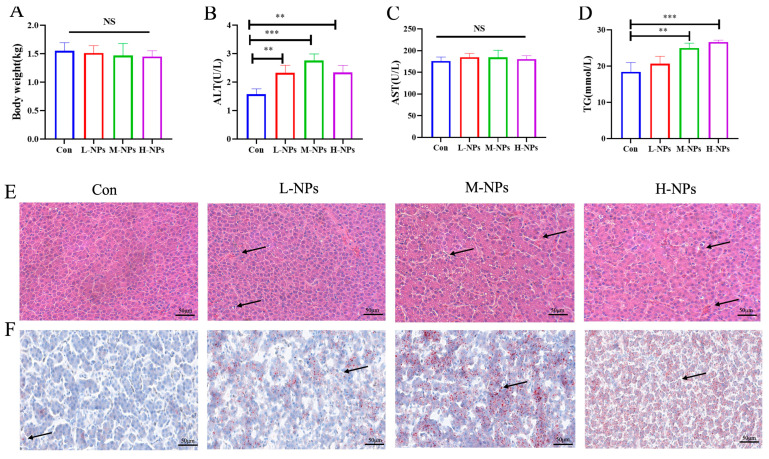
Effects of NPs exposure on growth performance and hepatic histopathology in chickens. (**A**) Body weight changes in chickens following NPs exposure. (**B**) The content of ALT in the serum in four groups. (**C**) The content of AST in the serum in four groups. (**D**) The content of TG in the serum in four groups. (**E**) Representative hematoxylin and eosin (H&E)-stained liver sections showing histological alterations (Black arrows indicate disorganized hepatic cords and blood extravasation, scale bar: 50 μm). (**F**) Oil Red O (ORO)-stained liver sections demonstrating lipid accumulation (Black arrow indicates lipid droplets, scale bar: 50 μm). All values of (**A**–**D**) are means ± SD; *n* = 6 per group, with ** *p <* 0.01, *** *p <* 0.001 determined by one-way ANOVA. “ns” is “no significant”.

Chickens were exposed to NPs via feed mixture administration for 120 days. No significant changes in body weight were observed (Figure 2A). Additionally, serum levels of ALT, a marker of liver function, were significantly elevated compared to the con group (Figure 2B), while no significant difference occurred in AST (Figure 2C). Histopathological analyses revealed hepatocellular inflammatory infiltrates, blood extravasation, and disorganization of hepatic cords in NP-exposed chickens, with severity increasing from the M-NPs group onward (Figure 2E). TG levels exhibited a dose-dependent increase (Figure 2D). Similarly, Oil Red O staining demonstrated significantly increased lipid droplet accumulation following NP exposure (Figure 2F). These results indicate that the L-NPs group exhibited relatively milder degrees of hepatic injury and lipid accumulation. Given that significant hepatic injury and lipid accumulation were observed starting from the M-NPs group, the M-NPs and con groups were selected for subsequent metabolomic analysis.

### 3.3. Metabolite Profiling with Data Analysis of Chicken Liver

**Figure 3:**
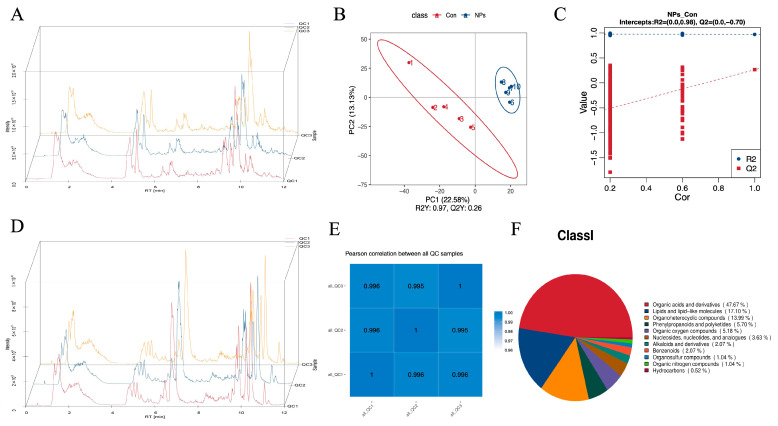
Metabolite profiling and QC analysis in chicken liver. (**A**) TIC chromatogram of metabolites in chicken liver (positive ion mode; *y*-axis: intensity). (**B**) PLS-DA score plot. (**C**) PLS-DA valid plot. (**D**) TIC chromatogram of metabolites in chicken liver (negative ion mode; *y*-axis: intensity). (**E**) Correlation analysis of QC samples for liver metabolites. (**F**) Pie chart showing classification and quantities of detected metabolites.

The total ion current (TIC) of the quality control (QC) samples was compared with the overlapping spectra. The response intensity and retention time of each chromatographic peak overlapped, indicating that there was little variation caused by instrument error throughout the whole experiment, and the data quality was reliable (Figure 3A,D). After LC-MS/MS analysis, we detected 49,552 features in positive ion mode and 48,793 in negative ion mode. Applying a coefficient of variation (CV) threshold of <30%, we identified a total of 1305 positive ion mode metabolites, and 913 negative ion mode metabolites were identified. QC samples exhibited near-ideal correlation values (close to 1), confirming excellent system stability and data reliability (Figure 3E). Multivariate analysis revealed clear metabolic distinctions between groups. Partial least squares discriminant analysis (PLS-DA) showed that more than 50% of the variance was explained by the first two coordinates, indicating a robust model. Samples clustered within 95% confidence intervals, with distinct separation between the Con and NPs groups (Figure 3B,C).

**Figure 4:**
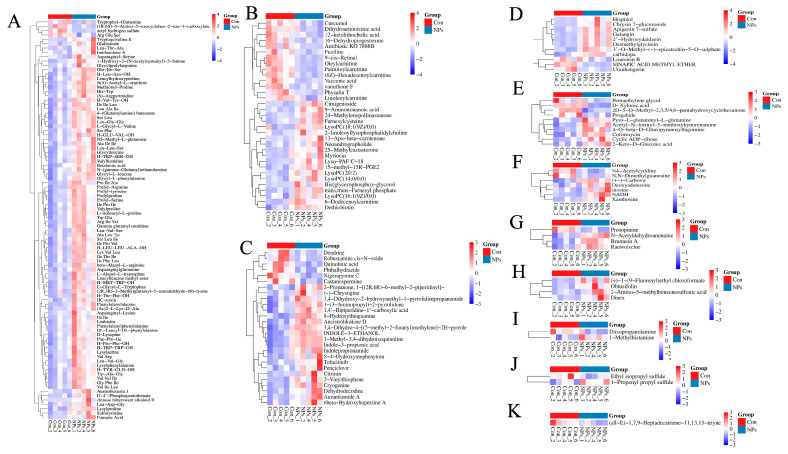
Heatmap of the abundance of the 193 metabolites belonging to 11 categories. (**A**) Organic acids and derivatives. (**B**) Lipids and lipid-like molecules. (**C**) Organoheterocyclic compounds. (**D**) Phenylpropanoids and polyketides. (**E**) Organic oxygen compounds. (**F**) Nucleosides, nucleotides, and analogues. (**G**)Alkaloids and derivatives. (**H**) Benzenoids. (**I**) Organosulfur compounds. (**J**) Organic nitrogen compounds. (**K**) Hydrocarbons.

Based on the set threshold (VIP > 1.0, 0.667 < FC > 1.5, and p value < 0.05). Qualitative and quantitative analyses were conducted on metabolites in chicken liver samples from the Con and NPs groups. One hundred and ninety-three metabolites were identified and divided into 11 categories: Organic acids and derivatives, Lipids and lipid-like molecules, Organoheterocyclic compounds, Phenylpropanoids and polyketides, Organic oxygen compounds, Nucleosides, nucleotides, and analogs, Alkaloids and derivatives, Benzenoids, Organosulfur compounds, Organic nitrogen compounds, and Hydrocarbons (Figure 3F). Organic acids and derivatives were the most abundant metabolites, with 92 observed, accounting for 47.67% of all metabolites (Figure 3F and Figure 4A). Lipids and lipid-like molecules were the second most abundant metabolites, with 33 detected, accounting for 17.1% of all metabolites (Figure 3F and Figure 4B). Organoheterocyclic compounds were the third most abundant metabolites, with 27 detected, accounting for 13.99% of all metabolites (Figure 3F and Figure 4C). We found that metabolites such as Phenylalanylalanine, 9-cis-Retinaland 8-Hydroxythioguanine clustered the samples into two groups. Figure 4 displays the abundance of the 193 metabolites belonging to 11 categories.

### 3.4. KEGG Classification and Differential Metabolite Analysis of Chicken Liver

**Figure 5:**
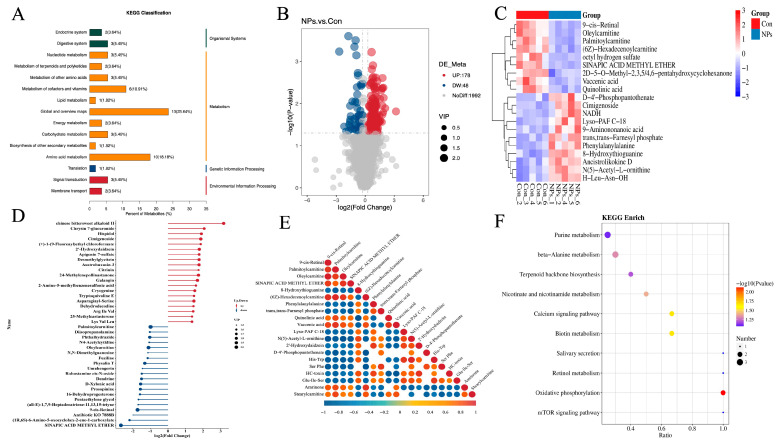
Multimodal Analysis of Differential Metabolites. (**A**) KEGG pathway hierarchy (Left axis: secondary class; Right axis: primary class). (**B**) Volcano plot of differential metabolites (Red: upregulated, Blue: downregulated; Dot size: VIP). (**C**) Hierarchical clustering dendrogram of differential metabolites. (**D**) Stick figure plot of differential metabolites (Red bars: upregulation; Blue bars: downregulation; Bar length: |log_2_FC|; Terminal dot size: VIP). (**E**) Analysis of Correlation with Differentially Metabolites (top 20; Red: +1; Blue: −1; Gray: *p* > 0.05). (**F**) KEGG enrichment bubble plot (top 10; Bubble size: differential metabolite count).

**Figure 6:**
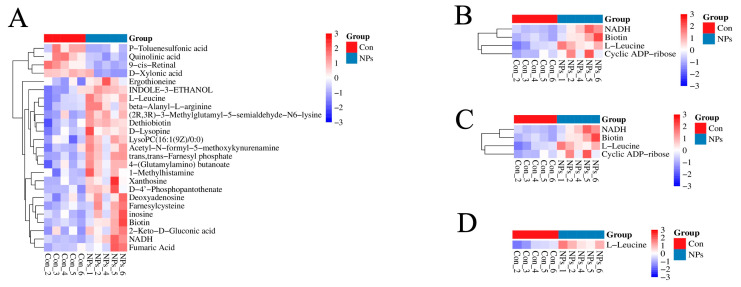
Heatmap of KEGG-classified metabolites. (**A**) Metabolites Classified under Metabolism of KEGG-classified metabolites. (**B**) Metabolites Classified under Environmental Information Processing of KEGG-classified metabolites. (**C**) Metabolites Classified under Organismal Systems of KEGG-classified metabolites. (**D**) Metabolites Classified under Genetic Information Processing of KEGG-classified metabolites.

**Figure 7:**
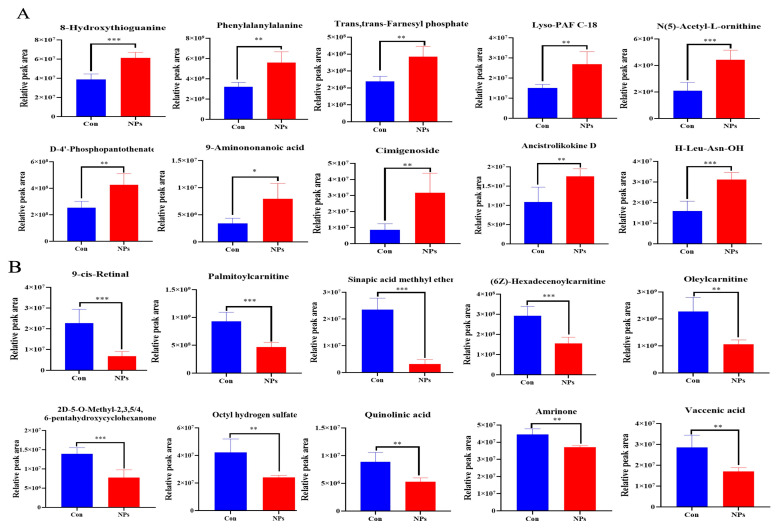
Differential metabolites of the Con and NPs groups. (**A**) The peak area of representative key metabolites was upregulated. (**B**) The peak area of representative key metabolites was downregulated. All values of (**A**,**B**) are means ± SD; *n* = 6 per group, with * *p* < 0.05, ** *p* < 0.01, *** *p* < 0.001 determined by Student’s *t*-test.

To further characterize the metabolites distinguishing the Con and NPs groups, a KEGG classification diagram of the differential metabolites was generated. Metabolism represented the most abundant KEGG category, comprising 80% of the assigned KO pathways, including Global and overview maps, Amino acid metabolism, Metabolism of cofactors and vitamins, among others (Figure 5A and Figure 6A, Table 2). Environmental Information Processing was the second most KEGG classified metabolite category, accounting for 9.09% of the KO Pathway, including Membrane transport, Signal transduction (Figure 5A and Figure 6B, Table 2). Organismal Systems also accounted for 9.09% of the KO Pathway, including the Digestive system and the Endocrine system (Figure 5A and Figure 6C, Table 2). Genetic Information Processing was the least, accounting for 1.82% of the KO Pathway, including Translation (Figure 5A and Figure 6D, Table 2). L-leucine and NADH function as pivotal metabolic hubs, participating in five and three pathways, respectively (Figure 6, Table 2). DAMs were identified using FC > 1.2 and *p* < 0.05. The DAMs between the Con and NPs groups were visualized by a volcano plot (Figure 5B). In total, 226 metabolites were identified by comparing Con and NPs samples, including 178 upregulated and 48 downregulated metabolites (Table S1). To better assess the metabolic profiles exhibiting significant differences between the Con and NPs groups, the top 20 differentially expressed metabolites were selected based on VIP values (Figure 5C). Among these, 10 of the differentially expressed metabolites were upregulated (Figure 7A), and 10 were downregulated (Figure 7B). To characterize the observed metabolic alterations, differential metabolites were systematically analyzed through multiple analytical approaches. Matchstick plots were employed to visualize the top 20 most significantly up- and down-regulated compounds, ranked by log2 FC (Figure 5D). Among the significantly upregulated metabolites were 1, Chinese bittersweet alkaloid II, Chrysin 7-glucuronide, and Hispidol, among others. Conversely, Sinapic acid methyl ether, Prosopinine, and Pentaethylene glycol were notably downregulated. The correlation diagram of differential metabolites shows that 9-cis-Retinal, Palmitoylcarnitine, and Oleylcarnitine are positively correlated, while N (5)-Acetyl-L-ornithine, His-Trp, Ser Phe, etc., are negatively correlated (Figure 5E). Furthermore, KEGG pathway analysis reveals that the Con and NPs groups were enriched in Purine metabolism, beta-Alanine metabolism, Terpenoid backbone biosynthesis, Retinol metabolism, and Oxidative phosphorylation (Figure 5F).

### 3.5. GSEA Function Enrichment Analysis of Chicken Liver

**Figure 8:**
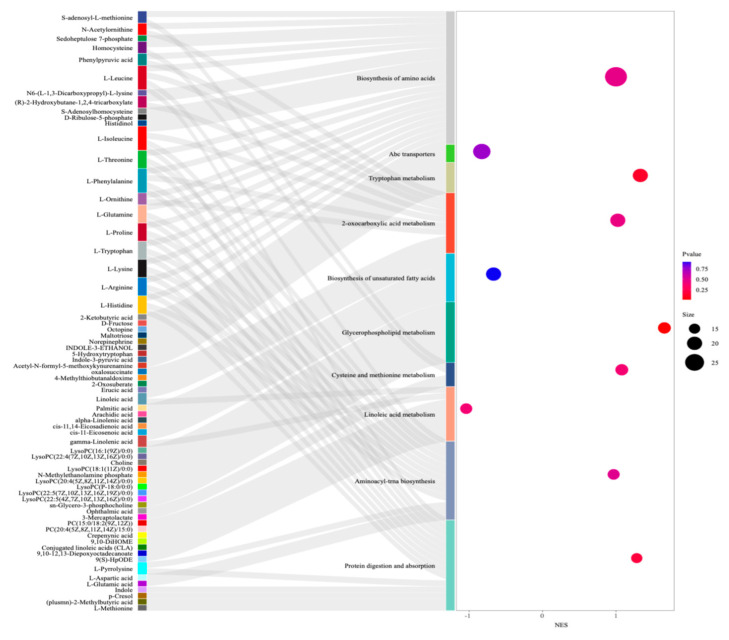
GSEA enrichment analysis of metabolic pathways. Sankey diagram of the KEGG pathways of metabolic enrichment in the liver of the Con and NPs groups. (Bubble size: number of differentially abundant metabolites per pathway).

To investigate the core metabolic enrichment in the Con and NPs groups, we performed biological process enrichment via GSEA. The analysis revealed that the following were enriched in the Con and NPs groups: biosynthesis of amino acids, ABC transporters, tryptophan metabolism, biosynthesis of unsaturated fatty acids, 2-oxocarboxylic acid metabolism, glycerophospholipid metabolism, cysteine and methionine metabolism, linoleic acid metabolism, aminoacyl-trna biosynthesis, and protein digestion and absorption (Figure 8 and Table S2). These results suggested that these metabolic pathways and core metabolic enrichments played significant roles in NPs exposure.

## 4. Discussion

The increasing prevalence of NPs contamination in agricultural systems demands thorough evaluation of their toxic effects, particularly in poultry, which plays a central role in global protein supply. While hepatotoxic effects of NPs have been established in various aquatic and mammalian models [20], their impact on poultry remains underexplored. This study addressed this knowledge gap by assessing liver pathology and metabolic alterations following chronic exposure to polystyrene NPs in chickens.

Consistent with reports of NPs biodistribution in mammalian models [21], monodisperse 100 nm NPs significantly accumulated in hepatic tissue. Following chronic exposure (120 days) to environmentally relevant doses (1, 10, 100 mg/kg bw), the following effects were observed: Histopathological lesions, including hepatocellular vacuolization and inflammatory infiltration. NPs exposure significantly increased serum aminotransferase activity in chickens, as indicated by our findings. Hamed et al. (2019) who observed a significant increase in juvenile O. niloticus when exposed in aquariums with dosages of 10 and 100 mg/L of microplastic [22]. Banaee et al. (2019) reported an increase in ALT in C. carpio after contact with polyethylene microplastic at a dosage of 250 and 500 µg/L [23]. It has been previously reported in the literature that exposure to polypropylene-MPs resulted in significant alterations to lipid metabolic profiles, with notable increases in triglyceride, fatty acid, free fatty acid, and lysophosphatidylcholine content [24]. We observed dose-dependent hepatic steatosis, which was confirmed through Oil Red O staining and increased serum triglyceride (TG) levels.

Metabolomics has become a core technology for revealing metabolic dynamics in organisms due to its high sensitivity and high-throughput analysis. As a new discipline, metabolomics performs qualitative and quantitative analysis of all small-molecule metabolites within cells of a biological system during specific periods and under defined conditions, aiming to quantitatively describe all endogenous metabolites of organisms and their responses to internal and external changes. The fundamental characteristics of metabolomics include high throughput, high sensitivity, and high stability in both analytical processes and database management. Furthermore, it has been extensively applied in medicine, food science, animal husbandry and veterinary science, botany, and other research fields [25,26]. The robustness of our metabolomic workflow was unequivocally validated through stringent measures. TIC overlap analysis of QC samples demonstrated near-perfect alignment in chromatographic peak response intensities and retention times, confirming minimal instrumental variation (CV < 5%) throughout the analytical sequence—a critical prerequisite for large-scale metabolomic studies [27]. Following LC-MS/MS analysis, we detected 49,552 features in positive ion mode and 48,793 in negative ion mode. Application of a coefficient of variation threshold < 30% yielded 1305 high-confidence metabolites in positive mode and 913 in negative mode, effectively eliminating technical noise while preserving biological signals.

Using untargeted LC-MS/MS metabolomics, we detected 193 differentially abundant metabolites, primarily organic acids and lipids. The upregulated phenylalanine and alanine indicate amino acid metabolism dysregulation following NPs exposure, consistent with established particle-induced metabolic disturbances [28]. Elevated 9-cis-retinal demonstrates disruption of retinol metabolism, aligning with toxicant-mediated retinoid pathway alterations [29]. KEGG enrichment and GSEA further supported these findings, highlighting significant pathway disruptions in purine metabolism, oxidative phosphorylation, ABC transporters, and fatty acid biosynthesis [30].

Terpenoid backbone biosynthesis (linked to cholesterol homeostasis) [31] and oxidative phosphorylation (indicating mitochondrial dysfunction) [32] were significantly altered pathways. Integrated multi-pathway analysis identified L-leucine and NADH as keystone metabolites perturbed by NPs exposure. L-leucine depletion disrupted three interdependent pathways: valine/leucine/isoleucine degradation (affecting protein turnover), fatty acid biosynthesis, and mTOR signaling [33]. Meanwhile, NADH accumulation reflected mitochondrial dysfunction, driving electron transport chain overload, impaired purine salvage pathways, and reduced TCA cycle flux [34]. This metabolic convergence establishes L-leucine and NADH as critical mediators of NP-induced hepatotoxicity [31,32,33]. Elevated 9-cis-retinal levels indicate disrupted retinol metabolism, promoting hepatic lipid accumulation through fatty acid transport protein 2 (FATP2) overexpression. Similarly, Phenylalanylalanine accumulation signifies impaired Amino acid catabolism a process mechanistically connected to mitochondrial dysfunction in steatotic livers [34]. The co-occurrence of 9-cis-retinal elevation and hepatic lipid deposition suggests a contributory mechanism to NPs induced steatosis, warranting further mechanistic investigation.

Gene set enrichment analysis (GSEA) is a powerful method that allows for the identification of coordinated changes in groups of genes, which can identify subtle but coordinated changes in gene expression that may be missed by other methods [35]. GSEA superiority over ORA in detecting coordinated pathway alterations, particularly: ABC transporters, Biosynthesis of unsaturated fatty acids, and Protein digestion and absorption. These findings corroborate transcriptomic studies demonstrating NPs inhibition of nutrient transport proteins [36,37,38,39]. Notably, the glycerophospholipid dysregulation aligns with recent mechanistic studies where lipid metabolism perturbations (specifically CD36-mediated pathways) directly drive hepatic steatosis pathogenesis, as demonstrated in CRISPR-Cas9-based NAFLD interventions targeting Rubicon [40].

This study establishes specific biomarkers for monitoring NPs contamination in poultry products. The significant reduction in hepatic essential amino acids observed in NPs groups, combined with GSEA, confirmed dysregulation of protein digestion pathways, collectively demonstrating that NPs compromise poultry product nutritional value through disruption of amino acid metabolic homeostasis. Consequently, these findings warrant assessment of NPs exposure risks to food safety standards and nutritional quality assurance in poultry-derived foods.

## 5. Conclusions

This study provides compelling evidence that chronic exposure to NPs induces significant hepatotoxicity in chickens, manifested as histopathological liver injury, serum biochemical analysis, and lipid accumulation. Metabolomic analyses revealed substantial disruptions in hepatic metabolic pathways, particularly involving purine metabolism, oxidative phosphorylation, and ABC transporter activity. The identification of key metabolites such as L-leucine, NADH, 9-cis-retinal, and phenylalanyl phenylalanine as central metabolic hubs underscores the systemic impact of NPs on liver function. These findings highlight the potential food safety risks associated with NPs contamination in poultry farming and emphasize the urgent need for regulatory measures and further research to mitigate NPs exposure in agricultural ecosystems.

## Figures and Tables

**Table 1:** The contents of C, N, and O in NPs.

Map Sum Spectrum		
Element	Wt%	Atomic%
C	89.96	91.64
N	6.3	5.51
O	3.73	2.86
Total	100	100

**Table 2:** List of the KEGG significantly unique metabolites between in Con and NPs groups.

KO Pathway Level 1	KO Pathway Level 2	Metabolites	FC	log2FC	*p* Value	VIP	Trend
Metabolism	Amino acid metabolism	Ergothioneine	1.26	0.33	0.032	1.798	up
		4-(Glutamylamino) butanoate	1.53	0.62	0.028	1.652	up
		D-Lysopine	1.98	0.98	0.026	1.649	up
		Fumaric Acid	2.46	1.3	0.044	1.468	up
		Quinolinic acid	0.59	−0.8	0.002	2.037	down
		L-Leucine	1.38	0.47	0.004	1.884	up
		(2R,3R)-3-Methylglutamyl- 5-semialdehyde-N6-lysine	1.67	0.74	0.01	1.793	up
		Acetyl-N-formyl-5- methoxykynurenamine	1.54	0.62	0.021	1.644	up
		1-Methylhistamine	1.7	0.77	0.043	1.539	up
		INDOLE-3-ETHANOL	2.18	1.12	0.025	1.737	up
	Biosynthesis of other secondary metabolites	Xanthosine	1.56	0.64	0.029	1.583	up
	Carbohydrate metabolism	2-Keto-D-Gluconic acid	1.52	0.6	0.037	1.571	up
		D-Xylonic acid	0.34	−1.6	0.024	1.803	down
		Fumaric Acid	2.46	1.3	0.044	1.468	up
	Energy metabolism	Fumaric Acid	2.46	1.3	0.044	1.468	up
		NADH	2.27	1.19	0.007	1.982	up
	Global and overview maps	4-(Glutamylamino) butanoate	1.53	0.62	0.028	1.652	up
		P-Toluenesulfonic acid	0.71	−0.5	0.015	1.854	down
		Biotin	1.42	0.51	0.013	1.829	up
		Fumaric Acid	2.46	1.3	0.044	1.468	up
		Xanthosine	1.56	0.64	0.029	1.583	up
		Quinolinic acid	0.59	−0.8	0.002	2.037	down
		Deoxyadenosine	1.7	0.76	0.036	1.544	up
		L-Leucine	1.38	0.47	0.004	1.884	up
		2-Keto-D-Gluconic acid	1.52	0.6	0.037	1.571	up
		NADH	2.27	1.19	0.007	1.982	up
		Dethiobiotin	1.86	0.89	0.042	1.639	up
		D-4′-Phosphopantothenate	1.68	0.75	0.003	1.988	up
		inosine	2.01	1.01	0.036	1.584	up
	Lipid metabolism	LysoPC(16:1(9Z)/0:0)	1.72	0.79	0.038	1.569	up
	Metabolism of cofactors and vitamins	Biotin	1.42	0.51	0.013	1.829	up
		9-cis-Retinal	0.3	−1.7	0.0003	2.065	down
		D-4′-Phosphopantothenate	1.68	0.75	0.003	1.988	up
		Dethiobiotin	1.86	0.89	0.042	1.639	up
		Fumaric Acid	2.46	1.3	0.044	1.468	up
		Quinolinic acid	0.59	−0.8	0.002	2.037	down
	Metabolism of other amino acids	beta-Alanyl-L-arginine	1.92	0.94	0.019	1.65	up
		D-4′-Phosphopantothenate	1.68	0.75	0.003	1.988	up
		Quinolinic acid	0.59	−0.8	0.002	2.037	down
	Metabolism of terpenoids and polyketides	Farnesylcysteine	2.39	1.26	0.028	1.549	up
		trans,trans-Farnesyl phosphate	1.61	0.69	0.002	1.959	up
	Nucleotide metabolism	inosine	2.01	1.01	0.036	1.584	up
		Xanthosine	1.56	0.64	0.029	1.583	up
		Deoxyadenosine	1.7	0.76	0.036	1.544	up
Environmental Information Processing	Membrane transport	Biotin	1.42	0.51	0.013	1.829	up
		L-Leucine	1.38	0.47	0.004	1.884	up
	Signal transduction	Cyclic ADP-ribose	1.94	0.96	0.019	1.625	up
		L-Leucine	1.38	0.47	0.004	1.884	up
		NADH	2.27	1.19	0.007	1.982	up
Organismal Systems	Endocrine system	Cyclic ADP-ribose	1.94	0.96	0.019	1.625	up
		NADH	2.27	1.19	0.007	1.982	up
	Digestive system	Cyclic ADP-ribose	1.94	0.96	0.019	1.625	up
		Biotin	1.42	0.51	0.013	1.829	up
		L-Leucine	1.38	0.47	0.004	1.884	up
Genetic Information Processing	Translation	L-Leucine	1.38	0.47	0.004	1.884	up

## Data Availability

The original contributions presented in this study are included in the article/Supplementary Material. Further inquiries can be directed to the corresponding authors.

## Supplementary Materials

The following supporting information can be downloaded at: https://www.mdpi.com/article/10.3390/foods14162781/s1, Table S1: List of the significant unique metabolites between Con and NPs groups. Table S2. KEGG pathways enriched by GSEA and their core enrichment metabolites in the Con and NPs groups.
