# Supplementary material for: Untargeted Metabolomics Uncovers Food Safety Risks: Polystyrene Nanoplastics Induce Metabolic Disorders in Chicken Liver
Source: Foods. 2025 Aug 10;14(16):2781. doi: 10.3390/foods14162781 (PMC12385707; doi:10.3390/foods14162781)
Supplement: Supplementary file 1 [file foods-14-02781-s001.zip › foods-3756290-supplementary.pdf]

Table S1. List of the significant unique metabolites between the Con and NPs groups.

Name	Formula	FC	log2FC	Pvalue	VIP	Trend
9-cis-Retinal	C20H28O	0.2999	-1.737	0.00025	2.065	down
Palmitoylcarnitine	C23H46NO4+	0.5032	-0.991	0.00032	2.098	down
Oleylcarnitine	C25H48NO4	0.463	-1.111	0.00045	2.055	down
SINAPIC ACID METHYL ETHER	C12H14O5	0.1556	-2.684	0.00059	2.047	down
8-Hydroxythioguanine	C5H5N5OS	1.579	0.659	0.0006	2.163	up
(6Z)-Hexadecenoylcarnitine	C23H43NO4	0.5316	-0.912	0.00072	2.03	down
Phenylalanylalanine	C12H16N2O3	1.7451	0.803	0.00124	1.993	up
trans,trans -Farnesyl phosphate	C15H27O4P	1.6137	0.69	0.00154	1.959	up
Quinolinic acid	C7H5NO4	0.5943	-0.751	0.00189	2.037	down
Vaccenic acid	C18H34O2	0.5982	-0.741	0.00215	1.995	down
Lyso-PAF C-18	C26H56NO6P	1.7772	0.83	0.00236	1.976	up
N(5)-Acetyl-L-ornithine	C7H14N2O3	2.1194	1.084	0.00241	1.961	up
2'-Hydroxydaidzein	C15H10O5	3.4657	1.793	0.00248	1.894	up
D-4'-Phosphopantothenate	C9H18NO8P	1.6787	0.747	0.00252	1.988	up
His-Trp	C17H19N5O3	2.3865	1.255	0.00295	1.905	up
Ser Phe	C12H16N2O4	1.7351	0.795	0.00299	1.877	up
HC-toxin	C21H32N4O6	1.5241	0.608	0.00322	1.92	up
Glu-Ile-Ser	C14H25N3O7	1.5054	0.59	0.00332	1.904	up
Amrinone	C10H9N3O	0.8306	-0.268	0.00339	2.036	down
Stearylcarnitine	C25H50NO4	0.721	-0.472	0.00355	1.924	down
L-Leucine	C6H13NO2	1.3811	0.466	0.00367	1.884	up
Isoferulic acid	C10H10O4	0.7596	-0.397	0.00378	1.911	down
Ala Ile Ile	C15H29N3O4	1.8769	0.908	0.00389	1.85	up
Leu-Val-Ser	C14H27N3O5	1.8568	0.893	0.00396	1.889	up
9-Aminononanoic acid	C9H19NO2	2.3122	1.209	0.00404	1.962	up
Nigerapyrone C	C13H14O4	0.5523	-0.857	0.00409	1.84	down
Cimigenoside	C35H56O9	3.6673	1.875	0.00421	1.929	up
Desmethylglycitein	C15H10O5	3.4333	1.78	0.00433	1.845	up

24-Methylenepollinastanone	C29H46O	3.3475	1.743	0.00442	1.838	up
Ancistrolikokine D	C24H25NO4	2.0074	1.005	0.00447	1.953	up
H-Leu-Asn-OH	C10H19N3O4	1.958	0.969	0.00453	1.951	up
Galangin	C15H10O5	3.2144	1.685	0.0049	1.829	up
(S)-Argpyrimidine	C11H18N4O3	2.0988	1.07	0.00574	1.829	up
octyl hydrogen sulfate	C8H18O4S	0.5699	-0.811	0.00617	1.978	down
Leucylhydroxyproline	C11H20N2O4	1.5157	0.6	0.00657	1.84	up
NADH	C21H29N7O14P2	2.274	1.185	0.00699	1.982	up
Hispidol	C15H10O4	3.7604	1.911	0.00731	1.769	up
N-Acetyldehydroanonaine	C19H15NO3	1.7507	0.808	0.00753	1.855	up
L-Alanyl-L-tryptophan	C14H17N3O3	1.7344	0.794	0.00766	1.871	up
Cryogenine	C26H29NO5	2.8953	1.534	0.00771	1.769	up
Ala Leu Tyr	C18H27N3O5	2.0961	1.068	0.00859	1.78	up
H-TYR-GLN-OH	C14H19N3O5	2.085	1.06	0.00874	1.856	up
Aurantiomide A	C19H24N4O4	1.5043	0.589	0.00878	1.807	up
Physalin T	C28H34O11	0.4003	-1.321	0.00892	1.873	down
Phthalhydrazide	C8H6N2O2	0.4875	-1.036	0.00903	1.848	down
Ile Thr Ile	C16H31N3O5	1.9548	0.967	0.00915	1.808	up
2D-5-O-Methyl-2,3,5/4,6 -pentahydroxycyclohexanone	C7H12O6	0.5553	-0.849	0.00953	1.938	down
(2R,3R)-3-Methylglutamyl -5-semialdehyde-N6-lysine	C12H23N3O4	1.675	0.744	0.00957	1.793	up
Ile Phe Leu	C21H33N3O4	2.0877	1.062	0.00992	1.847	up
Tyr-tyr	C18H20N2O5	1.4845	0.57	0.01002	1.726	up
Brunnein A	C13H12N2O3	2.1279	1.089	0.01049	1.821	up
LysoPC(20:2)	C28H54NO7P	1.8964	0.923	0.01077	1.753	up
Leu-Gly-Gly	C10H19N3O4	2.2681	1.181	0.01095	1.846	up
Methionyl-Proline	C10H18N2O3S	1.695	0.761	0.01099	1.831	up

Apigenin 7-sulfate	C15H10O8S	3.4398	1.782	0.01117	1.73	up
Trp-Ala-Gly	C16H20N4O4	1.7661	0.821	0.01134	1.809	up
Prosopinine	C16H33NO3	0.3357	-1.575	0.01141	1.744	down
Imidazooxazole	C4H3N3O	1.3459	0.429	0.01141	1.706	up
PC(P-16:0/20:2(11Z,14Z))	C44H84NO7P	1.3123	0.392	0.01152	1.85	up
Gly Phe Ile	C17H25N3O4	1.9866	0.99	0.01204	1.761	up
H-GLU-VAL-OH	C10H18N2O5	1.7854	0.836	0.01206	1.699	up
H-MET-TRP-OH	C16H21N3O3S	2.0565	1.04	0.01217	1.718	up
L-Glycyl-L-Tryptophan	C13H15N3O3	2.2243	1.153	0.01221	1.751	up
Biotin	C10H16N2O3S	1.421	0.507	0.0126	1.829	up
Progabide	C17H16ClFN2O2	0.5712	-0.808	0.01286	1.8	down
Chrysin 7-glucuronide	C21H18O10	4.2004	2.071	0.01289	1.701	up
(Ac)2-L-Lys-D-Ala	C13H23N3O5	1.6045	0.682	0.01324	1.709	up
2-Propanone, 1-[(2R,6R) -6-methyl-2-piperidinyl]-	C9H17NO	1.6688	0.739	0.01351	1.75	up
H-LEU-LEU-ALA-OH	C15H29N3O4	1.9808	0.986	0.0136	1.767	up
Val Ile Leu	C17H33N3O4	1.9979	0.998	0.01421	1.709	up
H-TRP-SER-OH	C14H17N3O4	1.8028	0.85	0.01422	1.738	up
L-isoleucyl-L-proline	C11H20N2O3	1.7317	0.792	0.01455	1.81	up
N5-Methyl-L-glutamine	C6H12N2O3	1.6081	0.685	0.01465	1.67	up
P-Toluenesulfonic acid	C7H8O3S	0.709	-0.496	0.01469	1.854	down
4-O-beta-D-Glucopyranosylfagomine	C12H23NO8	1.9129	0.936	0.01539	1.674	up
chinese bittersweet alkaloid II	C18H30N2O3	9.0792	3.183	0.01544	1.822	up
(+)-1-(9-Fluorenyl) ethyl chloroformate	C16H13ClO2	3.6566	1.87	0.01612	1.648	up
1-Methyl-3,4-dihydroisoquinoline	C10H11N	2.327	1.218	0.01819	1.812	up
Leu-Thr-Ala	C13H25N3O5	1.7522	0.809	0.01833	1.67	up

Tofacitinib	C16H20N6O	1.5672	0.648	0.01835	1.652	up
Austrobuxusin J	C35H54O9	3.4247	1.776	0.01837	1.863	up
Leucylleucine methyl ester	C13H26N2O3	1.6474	0.72	0.01848	1.632	up
2-amino-1,3,4,5 -icosanetetrol	C20H43NO4	0.8308	-0.267	0.01852	1.633	down
Curcumol	C15H24O2	0.637	-0.651	0.01853	1.705	down
1-Pyrrolidineethanol	C6H13NO	0.761	-0.394	0.01868	1.735	down
Tryptoquivaline E	C22H18N4O5	2.8046	1.488	0.01897	1.631	up
beta-Alanyl-L-arginine	C9H19N5O3	1.9185	0.94	0.01924	1.65	up
Linoleoylcarnitine	C25H46NO4	0.6387	-0.647	0.01926	1.879	down
Ala-Leu-Val-Ser	C17H32N4O6	1.4887	0.574	0.01938	1.624	up
Lysylserine	C9H19N3O4	1.957	0.969	0.01938	1.631	up
Cyclic ADP-ribose	C15H21N5O13P2	1.9394	0.956	0.0194	1.625	up
Val Val Ile	C16H31N3O4	2.1664	1.115	0.01956	1.698	up
Indole-3-propionic acid	C11H11NO2	2.1948	1.134	0.0196	1.8	up
1H-Benzotriazole	C6H5N3	1.3838	0.469	0.01971	1.694	up
Dendrine	C19H29NO4	0.3509	-1.511	0.01979	1.731	down
Phenylalanylphenylalanine	C18H20N2O3	1.864	0.898	0.02049	1.627	up
1-(3-Aminopropyl) -2-pyrrolidone	C7H14N2O	2.0258	1.018	0.0205	1.759	up
Acetyl-N-formyl-5-methoxykynurenamine	C13H16N2O4	1.5394	0.622	0.02053	1.644	up
2-Vinylthiophene	C6H6S	2.3428	1.228	0.02063	1.616	up
Castanospermine	C8H15NO4	0.5427	-0.882	0.02068	1.68	down
Asparaginylglutamine	C9H16N4O5	2.1534	1.107	0.02078	1.639	up
Tetranor-PGD1	C16H26O5	0.7159	-0.482	0.02282	1.757	down
Fuziline	C24H39NO7	0.4484	-1.157	0.02302	1.625	down
Leu-Leu-Ser	C15H29N3O5	1.7062	0.771	0.02318	1.636	up
Valylproline	C10H18N2O3	1.6771	0.746	0.02321	1.707	up
12-ketolithocholic acid	C24H38O4	0.6002	-0.736	0.02366	1.59	down

Valylhistidine	C11H18N4O3	2.5501	1.351	0.02413	1.692	up
D-Xylonic acid	C5H10O6	0.3387	-1.562	0.02418	1.803	down
Leuhistin	C11H19N3O3	1.6695	0.739	0.02428	1.61	up
Glycylleucine	C8H16N2O3	2.1402	1.098	0.02436	1.727	up
Thr Leu	C10H20N2O4	1.4692	0.555	0.02454	1.605	up
H-Thr-Phe-OH	C13H18N2O4	1.5026	0.588	0.02457	1.584	up
INDOLE-3-ETHANOL	C10H11NO	2.1806	1.125	0.02465	1.737	up
L-Glycyl-L-Valine	C7H14N2O3	2.1306	1.091	0.02577	1.704	up
2-Amino-5-methylbenzenesulfonic acid	C7H9NO3S	3.0303	1.599	0.02586	1.633	up
D-Lysopine	C9H18N2O4	1.977	0.983	0.0259	1.649	up
Asparaginyl-Serine	C7H13N3O5	2.7959	1.483	0.02635	1.853	up
N-(gamma-Glutamyl)ethanolamine	C7H14N2O4	1.6196	0.696	0.02652	1.663	up
Ile Pro Ile	C17H31N3O4	1.6143	0.691	0.02666	1.659	up
Phe-Pro-Ile	C20H29N3O4	1.6565	0.728	0.02683	1.632	up
(4E,6E)-3-hydroxydeca-4,6-dienoic acid	C10H16O3	0.7495	-0.416	0.02704	1.569	down
Ethyl isopropyl sulfide	C5H12S	0.6428	-0.638	0.02708	1.595	down
Dehydrodecodine	C25H27NO5	2.7459	1.457	0.02711	1.602	up
Leu-Asp-Gly	C12H21N3O6	1.8829	0.913	0.02711	1.572	up
Ile Ile Leu	C18H35N3O4	2.3156	1.211	0.02713	1.713	up
16-Dehydroprogesterone	C21H28O2	0.3302	-1.599	0.0273	1.589	down
15-methyl-15R-PGE2	C21H34O5	1.8354	0.876	0.02752	1.626	up
Citrinin	C13H14O5	3.3561	1.747	0.0276	1.565	up
Chrestifoline D	C28H22N2O3	1.4525	0.539	0.02783	1.551	up
4-(Glutamylamino) butanoate	C9H16N2O5	1.5322	0.616	0.02783	1.652	up
Leu-Val-Gly	C13H25N3O4	1.9045	0.929	0.02788	1.59	up
25-Methylcastasterone	C29H50O5	2.5977	1.377	0.02797	1.596	up
Arg Ile Val	C17H34N6O4	2.7162	1.442	0.02803	1.649	up
Farnesylcysteine	C18H31NO2S	2.3883	1.256	0.02811	1.549	up
Lys Val Leu	C17H34N4O4	2.5926	1.374	0.02839	1.669	up

Asparaginyl-Lysine	C10H20N4O4	2.4532	1.295	0.02859	1.552	up
Xanthosine	C10H12N4O6	1.5592	0.641	0.02862	1.583	up
Imidazolone A	C12H22N4O6	1.8734	0.906	0.02873	1.774	up
Betalamic acid	C9H9NO5	1.8108	0.857	0.02889	1.689	up
H-TRP-TRP-OH	C22H22N4O3	2.3662	1.243	0.0289	1.584	up
Rauwolscine	C21H26N2O3	1.5098	0.594	0.02908	1.613	up
6-Dodecenoylcarnitine	C19H35NO4	1.695	0.761	0.02933	1.559	up
Ser Leu Ile	C15H29N3O5	1.8277	0.87	0.02951	1.686	up
2-Oleoyl-sn-glycero-3-phosphocholine	C26H52NO7P	1.4842	0.57	0.02979	1.852	up
Val Phe Asp	C18H25N3O6	1.3229	0.404	0.02993	1.562	up
3'-O-Methyl-(-)-epicatechin-5-O-sulphate	C16H16O7S	2.3441	1.229	0.03001	1.612	up
Prolylproline	C10H16N2O3	1.6863	0.754	0.03006	1.61	up
3,4-Dihydroxy-2-hydroxymethyl-1-pyrrolidinepropanamide	C8H16N2O4	1.8694	0.903	0.03011	1.626	up
Tyrosyl-Valine	C14H20N2O4	1.4407	0.527	0.03012	1.61	up
Trp Glu	C16H19N3O5	2.2778	1.188	0.03054	1.676	up
Pyro-L-glutaminyl-L-glutamine	C10H15N3O5	2.0943	1.066	0.03065	1.568	up
Dinex	C12H14N2O5	2.0935	1.066	0.03078	1.651	up
carbidopa	C10H14N2O4	1.6115	0.688	0.03106	1.638	up
Coformycin	C11H16N4O5	1.894	0.921	0.0315	1.672	up
Benzetimide	C23H26N2O2	1.3764	0.461	0.03154	1.559	up
Ile Pro Val	C16H29N3O4	1.6895	0.757	0.03164	1.662	up
H-Val-Tyr-OH	C14H20N2O4	1.6317	0.706	0.03174	1.653	up
Glufosinate	C5H12NO4P	2.1368	1.095	0.03186	1.59	up
Glutarylcarnitine	C12H22NO6	1.4897	0.575	0.03187	1.767	up
Pentaethylene glycol	C10H22O6	0.3125	-1.678	0.03191	1.532	down
Ergothioneine	C9H15N3O2S	1.2564	0.329	0.03204	1.798	up
13-Oxo-9,11-octadecadienoic acid	C18H30O3	1.4768	0.563	0.03207	1.524	up
N4-Acetylcytidine	C11H15N3O6	0.4774	-1.067	0.03209	1.532	down

Penciclovir	C10H15N5O3	2.0049	1.004	0.03242	1.587	up
Pro Ile Ala	C14H25N3O4	1.7914	0.841	0.03315	1.55	up
Brunfelsamidine	C5H7N3	1.2438	0.315	0.03377	1.528	up
3-Methyl-N-phenylaniline	C13H13N	0.7692	-0.379	0.0338	1.514	down
Diisopropanolamine	C6H15NO2	0.4888	-1.033	0.03393	1.56	down
Prolyl-tyrosine	C14H18N2O4	1.6333	0.708	0.03407	1.602	up
1-Hydroxy-2-(N-acetylcysteinyl)-3-butene	C9H15NO4S	2.2763	1.187	0.03413	1.558	up
1-Propenyl propyl sulfide	C6H12S	1.5264	0.61	0.03416	1.55	up
4'-Hydroxy-3'-methylacetophenone	C9H10O2	0.7068	-0.501	0.03435	1.554	down
Lysylphenylalanine	C15H23N3O3	1.8639	0.898	0.03485	1.525	up
(1R,6S)-6-Amino-5-oxocyclohex-2-ene-1-carboxylate	C7H9NO3	0.2187	-2.193	0.03487	1.615	down
Ser Leu	C9H18N2O4	1.7828	0.834	0.035	1.638	up
Ile Ile	C12H24N2O3	1.5491	0.631	0.03526	1.527	up
Indolepropionamide	C11H12N2O	1.6604	0.732	0.03544	1.54	up
DL-Leucyl-DL-phenylalanine	C15H22N2O3	1.6769	0.746	0.0355	1.518	up
Obtusifolin	C16H12O5	2.3203	1.214	0.03553	1.508	up
Deoxyadenosine	C10H13N5O3	1.6988	0.764	0.03555	1.544	up
inosine	C10H12N4O5	2.0136	1.01	0.0357	1.584	up
Robustamine cis-N-oxide	C20H27NO5	0.3541	-1.498	0.03586	1.585	down
Myriocin	C21H39NO6	2.0234	1.017	0.03595	1.497	up
Vielanin A	C32H42O5	1.2348	0.304	0.03596	1.519	up
Dihydroartemisinic acid	C15H24O2	0.6032	-0.729	0.03612	1.534	down
(+)-cyclopentanecarboxylic acid	C11H16O5	0.73	-0.454	0.03613	1.54	down
(-)-Chrysogine	C10H10N2O2	1.6684	0.738	0.03708	1.551	up
2-Keto-D-Gluconic acid	C6H10O7	1.5207	0.605	0.03728	1.571	up
Gamma glutamyl ornithine	C10H19N3O5	2.5434	1.347	0.03818	1.619	up
LysoPC(16:1(9Z)/0:0)	C24H48NO7P	1.7233	0.785	0.03822	1.569	up
Leu Ala Ile	C15H29N3O4	2.0023	1.002	0.03897	1.645	up
Bis(glycerophospho)-glycerol	C9H22O13P2	2.3275	1.219	0.03911	1.608	up

Sulfotyrosine	C9H11NO6S	1.5366	0.62	0.03933	1.507	up
Lysylproline	C11H21N3O3	1.5429	0.626	0.03972	1.62	up
LysoPC(18:1(9Z)/0:0)	C26H52NO7P	1.5333	0.617	0.04002	1.727	up
Tryptophyl-Glutamine	C16H20N4O4	0.6634	-0.592	0.04009	1.499	down
S-4-Hydroxymephenytoin	C12H14N2O3	1.8591	0.895	0.04021	1.479	up
Glycyl-L-leucine	C8H16N2O3	2.1651	1.114	0.04022	1.656	up
N,N-Dimethylguanosine	C12H17N5O5	0.4531	-1.142	0.04039	1.519	down
Val Arg	C11H23N5O3	1.8607	0.896	0.04074	1.567	up
Umuhengerin	C20H20O8	0.3643	-1.457	0.04106	1.506	down
(9Z)-(7S,8S)-Dihydroxyoctadecenoic acid	C18H34O4	1.3924	0.478	0.04155	1.467	up
3,4-Dihydro-4-[(5-methyl-2-furanyl)methylene]-2H-pyrrole	C10H11NO	1.9331	0.951	0.04227	1.646	up
Arg Glu Ser	C14H26N6O7	0.5194	-0.945	0.04244	1.612	down
Dethiobiotin	C10H18N2O3	1.8585	0.894	0.04247	1.639	up
1-Methylhistamine	C6H11N3	1.7021	0.767	0.04252	1.539	up
13-Apo-beta-carotenone	C18H26O	1.5147	0.599	0.04333	1.61	up
(+-)-Carbovir	C11H13N5O2	2.1435	1.1	0.04393	1.635	up
Fumaric Acid	C4H4O4	2.4556	1.296	0.04398	1.468	up
1,4'-Bipiperidine-1'-carboxylic acid	C11H20N2O2	2.3421	1.228	0.0443	1.603	up
Loureirin B	C18H20O5	0.6173	-0.696	0.04433	1.547	down
Prolyl-Arginine	C11H21N5O3	1.6831	0.751	0.04435	1.59	up
(all-E)-1,7,9-Heptadecatriene-11,13,15-triyne	C17H18	0.3078	-1.7	0.0445	1.517	down
Cyclo(L-Leu-L-Pro)	C11H18N2O2	1.2481	0.32	0.04482	1.533	up
Iminodiacetic acid	C4H7NO4	1.2395	0.31	0.04486	1.5	up
Antibiotic KO 7888B	C16H22N2O4	0.2521	-1.988	0.04546	1.494	down
LysoPC(14:0/0:0)	C22H46NO7P	1.6596	0.731	0.04595	1.457	up
Neoandrographolide	C26H40O8	2.0802	1.057	0.04637	1.446	up
Glycyl-L-phenylalanine	C11H14N2O3	1.8236	0.867	0.04638	1.633	up

2-linoleoyllysophosphatidylcholine	C26H50NO7P	1.6427	0.716	0.04677	1.544	up
Apo-13-zeaxanthinone	C18H26O2	1.4363	0.522	0.04698	1.566	up
yanuthone F	C22H32O4	0.6244	-0.68	0.047	1.468	down
6beta-Hydroxyhuperzine A	C15H18N2O2	1.6338	0.708	0.04782	1.436	up
Prolyl-Serine	C8H14N2O4	1.6579	0.729	0.04925	1.545	up
Glycylprolylarginine	C13H24N6O4	2.4866	1.314	0.04928	1.439	up
H-Pro-Phe-OH	C14H18N2O3	1.6377	0.712	0.04973	1.53	up

Table S2. KEGG pathways enriched by GSEA and their core enrichment metabolites in the Con and NPs groups.

KEGG Term	Pvalue	ES	NES	Metabolites	Core enrichment
Biosynthesis of amino acids	0.464	0.3426	0.9945	S-adenosyl-L-methionine	Yes
				N-Acetylornithine	Yes
				Sedoheptulose 7-phosphate	Yes
				Homocysteine	Yes
				Phenylpyruvic acid	Yes
				L-Leucine	Yes
				N6-(L-1,3-Dicarboxypropyl)-L-lysine	Yes
				(R)-2-Hydroxybutane-1,2,4-tricarboxylate	Yes
				S-Adenosylhomocysteine	Yes
				D-Ribulose-5-phosphate	Yes
				Histidinol	Yes
				L-Isoleucine	Yes
				L-Threonine	Yes
				L-Phenylalanine	Yes
				L-Ornithine	Yes
				L-Glutamine	Yes
				L-Proline	Yes
				L-Tryptophan	Yes
				L-Lysine	Yes
				L-Arginine	Yes
				L-Histidine	Yes
				2-Ketobutyric acid	Yes
				L-Methionine	No
				Alpha-ketoisovaleric acid	No
				N-Acetyl-L-glutamate 5-semialdehyde	No

ABC transporters	0.706	-0.278	-0.842	Citric Acid	No
				O-Succinyl-L-homoserine	No
				o-Phospho-L-serine	No
				Phosphoenolpyruvic acid	No
				D-Fructose	Yes
				Octopine	Yes
				Maltotriose	Yes
				Thiamine	No
				Choline	No
				Biotin	No
				L-Leucine	No
				Betaine	No
				Spermidine	No
				4-Amino-5-hydroxymethyl -2-methylpyrimidine	No
				Xylitol	No
				L-Isoleucine	No
				L-Threonine	No
				L-Phenylalanine	No
				L-Ornithine	No
				Choline sulfate	No
				L-Glutamine	No
				L-Proline	No
				L-Lysine	No
				L-Arginine	No
				L-Histidine	No
				Glutathione	No
				Taurine	No

Tryptophan metabolism	0.092	0.5032	1.3531	Norepinephrine	Yes
				INDOLE-3-ETHANOL	Yes
				5-Hydroxytryptophan	Yes
				Indole-3-pyruvic acid	Yes
				Acetyl-N-formyl-5 -methoxykynurenamine	Yes
				Xanthurenic acid	No
				Indole	No
				2-Aminomuconic acid semialdehyde	No
				5-Hydroxyindole-3-acetic acid	No
				L-Tryptophan	No
				(Indol-3-yl) acetamide	No
				Indolelactic acid	No
				Serotonin	No
				Indole-3-acetaldehyde	No
				L-Kynurenine	No
				3-Methyldioxyindole	No
				5-Hydroxyindolepyruvate	No
				2-Formaminobenzoylacetate	No
				Formylanthranilate	No
				Quinolinic acid	No
2-oxocarboxylic acid metabolism	0.44	0.3799	1.0305	N-Acetylornithine	Yes
				oxalosuccinate	Yes

				Phenylpyruvic acid	Yes
				4-Methylthiobutanaldoxime	Yes
				L-Leucine	Yes
				(R)-2-Hydroxybutane -1,2,4-tricarboxylate	Yes
				L-Isoleucine	Yes
				L-Phenylalanine	Yes
				2-Oxosuberate	Yes
				L-Ornithine	Yes
				L-Tryptophan	No
				L-Lysine	No
				2-Ketobutyric acid	No
				L-Methionine	No
				Alpha-ketoisovaleric acid	No
				Citraconic acid	No
				cis-(Homo)3-aconitate	No
				N-Acetyl-L-glutamate 5-semialdehyde	No
				Citric Acid	No
				8-Methylthiooctanaldoxime	No
Biosynthesis of unsaturated fatty acids	0.915	-0.23	-0.674	Erucic acid	Yes
				Linoleic acid	Yes
				Palmitic acid	Yes
				Arachidic acid	Yes
				alpha-Linolenic acid	Yes
				cis-11,14-Eicosadienoic acid	Yes
				cis-11-Eicosenoic acid	Yes

				gamma-Linolenic acid	Yes
				cis-13,16-Docosadienoic acid	No
				cis-11,14,17-Eicosatrienoic acid	No
				homo-gamma-Linolenic acid	No
				Lignoceric acid	No
				Arachidonic acid	No
				cis-7,10,13,16,19-Docosapentaenoic acid	No
				cis-7,10,13,16-Docosic acidtraenoic acid	No
				cis-4,7,10,13,16,19-Docosahexaenoic acid	No
				Nervonic acid	No
				Behenic acid	No
				cis-5,8,11,14,17-Eicosapentaenoic acid	No
				Stearic acid	No
Glycerophospholipid metabolism	0.01	0.646	1.6706	LysoPC(16:1(9Z)/0:0)	Yes
				LysoPC(22:4(7Z,10Z,13Z,16Z)/0:0)	Yes
				Choline	Yes
				LysoPC(18:1(11Z)/0:0)	Yes
				N-Methylethanolamine phosphate	Yes
				LysoPC(20:4(5Z,8Z,11Z,14Z)/0:0)	Yes
				LysoPC(P-18:0/0:0)	Yes
				LysoPC(22:5(7Z,10Z,	Yes

				13Z,16Z,19Z)/0:0)	
				LysoPC(22:5(4Z,7Z,10Z, 13Z,16Z)/0:0)	Yes
				sn-Glycero-3-phosphocholine	Yes
				LysoPC(18:3(6Z,9Z,12Z)/0:0)	No
				Phosphodimethylethanolamine	No
				PC(20:4(5Z,8Z,11Z,14Z)/18:0)	No
				LysoPC(22:6(4Z,7Z,10Z, 13Z,16Z,19Z)/0:0)	No
				PC(15:0/18:2(9Z,12Z))	No
				PC(20:4(5Z,8Z,11Z,14Z)/15:0)	No
				O-Phosphorylethanolamine	No
Cysteine and methionine metabolism	0.358	0.422	1.0835	S-adenosyl-L-methionine	Yes
				Ophthalmic acid	Yes
				Homocysteine	Yes
				3-Mercaptolactate	Yes
				S-Adenosylhomocysteine	No
				L-2-Aminobutyric acid	No
				5'-Methylthioadenosine	No
				L-Aspartic acid	No
				2-Ketobutyric acid	No
				Glutathione	No
				L-Methionine	No
				N-formylmethionine	No
				L-Cysteic acid	No

				5'-S-Methyl-5'-thioinosine	No
				methionine sulfoxide	No
				O-Succinyl-L-homoserine	No
				o-Phospho-L-serine	No
Linoleic acid metabolism	0.377	-0.377	-1.059	PC(15:0/18:2(9Z,12Z))	Yes
				PC(20:4(5Z,8Z,11Z,14Z)/15:0)	Yes
				Linoleic acid	Yes
				Crepenynic acid	Yes
				9,10-DiHOME	Yes
				Conjugated linoleic acids (CLA)	Yes
				9,10-12,13-Diepoxyoctadecanoate	Yes
				gamma-Linolenic acid	Yes
				9(S)-HpODE	Yes
				13(S)-HODE	No
				homo-gamma-Linolenic acid	No
				alpha-Dimorphecolic acid	No
				Arachidonic acid	No
				9,10-Epoxy-13-hydroxy-11-octadecenoate	No
				PC(20:4(5Z,8Z,11Z,14Z)/18:0)	No
				9,10,13-TriHOME	No
Aminoacyl-trna biosynthesis	0.48	0.385	0.9934	L-Pyrrolysine	Yes
				L-Leucine	Yes
				L-Isoleucine	Yes

				L-Threonine	Yes
				L-Phenylalanine	Yes
				L-Glutamine	Yes
				L-Proline	Yes
				L-Tryptophan	Yes
				L-Aspartic acid	Yes
				L-Glutamic acid	Yes
				L-Lysine	Yes
				L-Arginine	Yes
				L-Histidine	Yes
				L-Methionine	No
				L-Tyrosine	No
				o-Phospho-L-serine	No
Protein digestion and absorption	0.216	0.5048	1.2269	L-Pyrrolysine	Yes
				L-Leucine	Yes
				Indole	Yes
				L-Isoleucine	Yes
				L-Threonine	Yes
				L-Phenylalanine	Yes
				p-Cresol	Yes
				L-Glutamine	Yes
				L-Proline	Yes
				L-Tryptophan	Yes
				L-Lysine	Yes
				L-Arginine	Yes
				L-Histidine	Yes
				(plusmn)-2-Methylbutyric acid	Yes

	L-Methionine	Yes
